# Prevalence of diarrheal illness and healthcare-seeking behavior by age-group and sex among the population of Gaza strip: a community-based cross-sectional study

**DOI:** 10.1186/s12889-019-7070-0

**Published:** 2019-06-07

**Authors:** Samer Abuzerr, Simin Nasseri, Masud Yunesian, Mahdi Hadi, Amir Hossein Mahvi, Ramin Nabizadeh, Ayman Abu Mustafa

**Affiliations:** 1 Department of Environmental Health Engineering, School of Public Health, International Campus, Tehran University of Medical Sciences, Tehran, Iran; 2 Department of Environmental Health Engineering, School of Public Health, Tehran University of Medical Sciences, Tehran, Iran; 3 Center for Water Quality Research (CWQR), Institute for Environmental Research (IER), Tehran University of Medical Sciences, Tehran, Iran; 4 Department of Research Methodology and Data Analysis, Institute for Environmental Research, Tehran University of Medical Sciences, Tehran, Iran; 5 Department of Research, Directorate General of Human Resources Development, Ministry of Health, Gaza Strip, Palestine

**Keywords:** Behavior, Diarrheal illness, Gaza strip, Healthcare seeking, Water source

## Abstract

**Background:**

In the Gaza strip, diarrhea is one of main reasons for children visiting primary healthcare centers. Hence, we investigate predictors of the diarrheal illness and health care-seeking behavior among different age groups.

**Methods:**

This community-based cross-sectional survey was conducted from August 2017 to June 2018 among 1857 households. A pretested structured questionnaire included information about socio-demographic, sanitation, hygiene, source of water, diarrheal illness, and seeking healthcare in households was administered to head of household. To achieve representativeness for the five Gaza’s governorates, a cluster random sampling was applied.

**Results:**

Of the 1857 household’s heads, 421 (22.7%) reported an episode of diarrhea during the 48 h preceding the interview resulting an overall prevalence rate of 3.8 per 100 individuals. The prevalence of diarrhea was statistical significant greater in males (5.4/100) compared to females (1.3/100) in all age groups (*p* <  0.05). Socio-demographic, economic, water, sanitation, and hygiene factors were predictors of the diarrheal illness and seeking of non-professional healthcare for diarrhea illness treatment among. A transition behavior from professional to non-professional and vice versa in seeking healthcare in each diarrheal episode was found.

**Conclusions:**

We recommend improving the status of water, sanitation, and hygiene in the Gaza strip’s households to reduce diarrhea among the population of Gaza strip. Community sensitization about the importance of seeking care at primary health centers because treatment of children is available for free or in low costs.

## Background

Diarrhea is the second worldwide most common cause of death in children below the 5syears old in particular. According to the world health organization (WHO), more than 760,000 children a year die as a consequence of diarrheal diseases [1]. Young children especially who are living in low-income states are at great risk for diarrheal consequences such as dehydration and malnutrition. Country-based investigations are highly relevant to identify diarrheal illness related-risk factors and inspect of healthcare-seeking behavior in society and recognize its implications on public health [2].

In the Gaza strip, diarrheal illness is the main cause for childhood visits to primary healthcare centers and hospitals [3]. Moreover, the United Nations relief and works agency for Palestine refugees in the Near East (UNRWA) revealed that the prevalence of diarrhea was 4017.1 case per 100,000 inhabitants in 2009, thereafter rose to 6448.2 cases per 100,000 inhabitants in 2013, resulted mainly from alter of water quality, sanitation facilities and hygiene practices (WASH) at households [4].

Factors influencing healthcare seeking behavior, include individual factors, accessibility to service, the severity of illness, the trust of healthcare providers, and prior beliefs concerning treatment of the illness [5–7]. Moreover, the presence of considerable social, economic and information constraints would limit the use of publicly-funded healthcare and seek professional healthcare [8].

Traditional practices at houses revealed improper treatment of diarrhea in which delay seeking health care has resulted in a remarkable number of mortalities [9, 10]. Therefore, understanding the constraints are crucial especially in poverty-stricken communities like in Gaza strip to mitigate the barriers and evaluate the effectiveness of such interventions [11].

The Palestinian National Authority is the main health insurance provider and allocates 37.7% of its annual budget to the health sector. The Palestinian ministry of health is responsible for providing health services to the Gazans. The majority of MOH funding comes from foreign aid and fees for health services [12]. The Ministry of Health has about 54 primary healthcare centers (PHCCs) and 13 hospitals distributed throughout Gaza strip governorates. Furthermore, the nongovernmental organizations (NGOs) and the private health sector play a vital role in the provision of third-care services, outpatient clinics, and rehabilitation centers through 8 PHCCs and 14 hospitals [13]. The UNRWA has 22 health centers which offer primary healthcare to Palestinian 1,167,572 refugees in the Gaza strip [12]. The surveillance system for communicable diseases in the Gaza strip remains limited due to lack of logistical capacity and financial resources.

Understanding risk factors associated with the occurrence of diarrhea in all age groups of the society and the predictors of seeking non-professional healthcare for diarrhea treatment may be helpful in reducing diarrheal morbidity and mortality. To the best of our knowledge, burdens of diarrheal diseases and health care-seeking behavior among the population at risk in Gaza strip have not been assessed. Thus, the aim of this study was to identify diarrheal illness related-risk factors and predictors for seeking nonprofessional care for diarrhea treatment among the population of Gaza strip. Moreover, the trend of seeking healthcare behavior from professional and non-professional healthcare providers in the last three diarrheal episodes was explored.

## Methods

### Study design and setting

This community-based cross-sectional study was carried out from 1st of August 2017 to 28th June 2018 in the Gaza strip. Gaza strip classified as the third most densely populated areas in the world and includes five governorates as follow: North Gaza, Gaza, Middle area, Khan Younis, and Rafah governorate [14].

### Sample size calculation

The sample size in this study was calculated using the following formula [15]:

1 $$ Sample\ size\ (n)=\frac{Z_{1-\alpha /2}^2\ P\left(1-P\right)}{d^2}=\frac{(1.96)^2(0.50)\ \left(1-0.50\right)}{(0.05)^2}=384 $$

Where, Z_1-α/2_ = Standard normal variate (Z value is 1.96 for a 95% confidence level). p = Response distribution (50%). d = Margin of error (5%).

The calculated sample size was 384 households. Unexpectedly, after data collection had finished, some items have gotten zero frequency at the sample size of 384 households. Consequently, we have recalculated the sample size after decrease margin of error (2.274%) to increase the sample size and raise the level of representation as well as to get a narrower confidence interval [16]. Eventually, the sample size calculated by the same equation with an adjusted margin of error was 1857 households.

2 $$ Sample\ size\ (n)=\frac{Z_{1-\alpha /2}^2\ P\left(1-P\right)}{d^2}=\frac{(1.96)^2(0.50)\ \left(1-0.50\right)}{(0.02274)^2}=1,857 $$

### Data collection and sampling

A cluster random sampling was applied to achieve representativeness from all Gaza’s governorates. The number of surveyed households in each governorate was determined based on the total number of households in each governorate which was as follows: 126 in North Gaza, 466 in Gaza, 472 in Middle area, 477 in Khan Younis, and 316 in Rafah governorate. Data were collected by four well-trained interviewers and 15 min were enough to complete the questionnaire by face to face interview.

### Study tool

A pretested structured questionnaire was administered to head of household and encompassed six parts [11]: socio-demographic (age, gender, housing area, monthly income, marital status, education level, etc.), sanitation status (4 items), hygiene status (1 item), sources of household water (3 items), diarrheal illness (4 items), and seeking healthcare (1 items), the participants were questioned about the professionalism of healthcare providers sought in the three preceding diarrheal episodes, as the selection of healthcare kind in each diarrheal episode might change. For categorical variables, the responses were measured on 2-point (yes/no) scale.

### Variable definitions

In our study, acute diarrhea was defined according to the World Health Organization (WHO), as the passage of three or more abnormally loose, watery, or liquid stools over a 24-h period [17]. Professional healthcare provider denotes medical personnel in primary healthcare centers, hospitals, or pharmacies, whereas non-professional provider indicates non-medical personnel such as traditional medicine therapist and in-home therapist. Primary education is considered low education. Improved water refers to treated water. Less than 1500 Israeli new shekel (INS), the local currency, is classified as low monthly income (1 USD ≈ 3.6 INS). Urban area is defined as a region with high population density and infrastructure of built environment, whereas rural area is defined as a region with low population density and small settlements. Agricultural areas are rural in nature.

### Statistical analysis

The Statistical Package for Social Science (SPSS) version 20 was used for data analysis. Descriptive statistics of frequency and percentage, and mean and standard deviation were performed for categorical and continuous variables respectively. The independent samples t-test was applied to investigate the differences between means. The chi-square test was used to determine the statistically significant differences between the different categorical variables. The difference in individual and household level characteristics between those who ‘reported diarrhea’ and those who presumably did not have diarrhea was examined using the Chi-square test. The odds ratio (OR) and the confidence interval (CI) for the risk factors associated with diarrheal illness and for seeking nonprofessional care for diarrhea treatment were reported using logistic regression analysis. A *p*-value < 0.05 was considered statistically significant.

## Results

### Socio-demographic characteristics

Altogether 1857 head of households were surveyed. The mean age ± SD of getting diarrhea was 15.6 ± 16.5 years old. About 611 (32.9%) lived in rural areas, while 1246 (67.1%) were from urban regions. Most of household’s heads were highly educated 1607 (86.5%). The vast majority of household’s heads were males 1621 (87.3%). Almost half of households lived with low income 908 (48.9%). Only 610 (32.8%) of households relied on non-improved water for drinking, whereas 1814 (0.98%) and 1022 (55%) used non-improved water for washing and cooking purposes, respectively (Table 1).

**Table 1:** Comparison of individual and household level characteristics between those who reported diarrhea’ and those who presumably did not have diarrhea

Variables	Did not get diarrhea *n* = 1436 (77%)	Got diarrhea *n* = 421 (23%)	*p*-value
Binary variables			
Housing area			
Rural	390 (27.2)	221 (52.5)	0.001
Urban	1046 (72.8)	200 (47.5)	
Marital status of the household head			
Married	1192 (83.0)	344 (81.7)	0.536
Single	244 (17.0)	77 (18.3)	
Education level of household head			
Less educated	147 (10.2)	103 (24.5)	0.001
High educated	1289 (89.8)	318 (75.5)	
Gender of household head			
Male	1255 (87.4)	366 (86.9)	0.803
Female	181 (12.6)	55 (13.1)	
Career of household head			
Craftsman	510 (35.5)	162 (38.5)	0.266
Public servant	926 (64.5)	259 (61.5)	
Monthly income of household			
Low income	740 (51.5)	168 (39.9)	0.001
High income	696 (48.5)	253 (60.1)	
House ownership			
Owned	1323 (92.1)	399 (94.8)	0.066
Rented	113 (7.9)	22 (5.2)	
Gender of person who had diarrhea			
Male	–	257 (61.0)	0.963
Female	–	164 (39.0)	
Source of drinking water			
Non improved	462 (32.2)	148 (35.2)	0.252
Improved	974 (67.8)	273 (64.8)	
Source of washing water			
Non improved	1405 (97.8)	409 (97.1)	0.407
Improved	31 (2.2)	12 (2.9)	
Source of cooking water			
Non improved	758 (52.8)	237 (56.3)	0.204
Improved	678 (47.2)	184 (43.7)	
Clean kitchen			
Yes	1244 (86.6)	128 (30.4)	0.001
No	192 (13.4)	293 (69.6)	
Sanitary toilet			
Yes	1170 (81.5)	14 (3.3)	0.001
No	266 (18.5)	407 (96.7)	
Kind of toilet			
Latrine with flush	960 (66.9)	31 (7.4)	0.001
Latrine without flush	476 (33.1)	390 (92.6)	
Place of wastewater disposal			
Open area around the house	232 (16.2)	304 (72.2)	0.001
Close sewerage system	1204 (83.8)	117 (27.8)	
Availability of soap for hands washing			
Yes	1078 (75.1)	189 (44.9)	0.001
No	358 (24.9)	232 (55.1)	
Continuous variables			
Age of household head (years)	41.3 ± 13.3 (22–67)	41.5 ± 14 (22–67)	0.801
Living in the area (years)	15.9 ± 10.5 (1–44)	15.5 ± 10.5 (1–44)	0.432
Number of rooms in house	2.7 ± 0.9 (1–5)	2.7 ± 0.8 (1–5)	0.612
Number of family Members	6.2 ± 1.7 (2–12)	6.2 ± 1.7 (2–12)	0.529
Age of getting diarrhea	–	15.6 ± 16.5 (1–67)	–
Number of children less than 5 in family	1.4 ± 0.7 (0–3)	1.5 ± 0.6 (0–3)	0.312
Distance to healthcare center (km)	1.6 ± 0.8 (1–4)	1.6 ± 0.8 (1–4)	0.916

For the continuous variables, the Mean ± SD (min-max) were presented instead of frequency and percentage as in the binary variables

Chi-square test was used to examine the difference between the two groups

### Prevalence of diarrheal disease

Four hundred twenty-one household head stated that there had been an episode of diarrhea within their household during the 48 h preceding the interview resulting in a prevalence rate of 3.8 per 100 individuals.

The prevalence rate of diarrhea among children ≤5 years old was 11.7 per 100 individuals, while it was 1.2 and 3.1 in the age groups between 6 and 15 years old and more than 16 years, respectively. The overall prevalence rate for all age groups was 3.8 per 100 individuals. With regards to gender differences, findings revealed statistical differences between males and females and prevalence of diarrhea was higher in males in all age groups; ≤ 5 years, 6–15 years, > 16 years (15.8, 2.1, and 4.2, respectively) (*P* <  0.001). By and large, diarrhea was more prevalent in males than females (5.4 and 1.3, respectively) (*p* <  0.001) (Table 2).

**Table 2:** The prevalence of diarrhea by age and sex groups among the population of Gaza strip

Age group (Years)	Male			Female			*P*-value	Al sexes		
	No. of individuals	Diarrheal cases	Rate/100	No. of individuals	Diarrheal cases	Rate/100		No. of persons	Diarrheal cases	Rate/100
≤5	1075	170	15.8	687	37	5.4	0.001	1762	207	11.7
6- ≤ 15	2064	43	2.1	1886	4	0.2	0.001	3950	47	1.2
≤15	3139	213	6.8	2573	41	1.6	0.001	5712	254	4.4
> 16	3658	153	4.2	1772	14	0.8	0.001	5430	167	3.1
All ages	6797	366	5.4	4345	55	1.3	0.001	11,142	421	3.8

### Independent factors associated with diarrhea illness

The logistic regression analysis showed that living in rural areas (OR = 2.1; 95% CI: 1.4–3.2), low education level of household’s head (OR = 2.7; 95% CI: 1.6–4.4), low monthly income (OR = 2.4; 95% CI: 1.7–3.5), having an unclean kitchen at home (OR = 1.4; 95% CI: 1.1–1.8), having non-sanitary toilet at home (OR = 278.6; 95% CI: 99.9–777.4), using a latrine without flush (OR = 2.5; 95% CI: 1.1–5.8), disposal of wastewater in open area around house (OR = 10.3; 95% CI: 6.9–15.5), and lack of soap for hands washing (OR = 3.7; 95% CI: 2.5–5.4), were predictors for developing diarrheal illness (Table 3).

**Table 3:** Predictors of diarrheal illness among the population of Gaza strip

Variable	Odds ratio	95% CI	*P*-value
Living in rural areas^a^	2.1	(1.4–3.2)	0.001
Low education level of household head^b^	2.7	(1.6–4.4)	0.001
Low monthly income of household^c^	2.4	(1.7–3.5)	0.001
Unclean kitchen^d^	1.4	(1.1–1.8)	0.045
Non-sanitary toilet^e^	278.6	(99.9–777.4)	0.001
Latrine without flush^f^	2.5	(1.1–5.8)	0.037
Disposal of wastewater in an open area around the house^g^	10.3	(6.9–15.5)	0.001
Lack of soap for hands washing^h^	3.7	(2.5–5.4)	0.001

The level of measurement for all variables in the table was nominal

The default references used for the variables in the table were:

^a^Living in urban areas

,^b^High education level of household head

^c^High monthly income of household

^d^Clean kitchen

^e^Sanitary toilet

^f^Latrine with flush

^g^Disposal of wastewater in a closed sewerage system

^h^Availability of soap for hands washing

### Healthcare seeking behavior

In bivariate analysis between those who sought healthcare from professional vs non-professional provider, we found significant differences and association with some independent variables. Education level, monthly income, and age of getting diarrhea are factors correlated with kind of healthcare seeking for treatment of diarrhea (*p* <  0.05) (Table 4).

**Table 4:** **|** Comparison of individual and household characteristics among people sought their medical care for diarrhea from professional and nonprofessional healthcare providers

Variables	Professional health provider *n* = 187 (44.5%)	A non-professional health service provider *N* = 233 (55.5%)	*P*-value
Housing area			0.637
Rural	96 (51.3)	125 (53.6)	
Urban	91 (48.7)	108 (46.4)	
Marital status of the household head			0.088
Married	146 (78.1)	197 (84.5)	
Single	41 (21.9)	36 (15.5)	
Education level of household head			< 0.001
Less educated	17 (9.1)	85 (36.5)	
High educated	170 (90.9)	148 (63.5)	
Gender of household head			0.109
Male	157 (84.0)	208 (89.3)	
Female	30 (16.0)	25 (10.7)	
Career of household head			0.166
Craftsman	79 (42.2)	83 (35.6)	
Public servant	108 (57.8)	150 (64.4)	
Monthly income of household			< 0.001
Low income	45 (24.1)	122 (52.4)	
High income	142 (75.9)	111 (47.6)	
House ownership			0.726
Owned	178 (95.2)	220 (94.4)	
Rented	9 (4.8)	13 (5.6)	
Clean kitchen			0.998
Yes	57 (30.5)	71 (30.5)	
No	130 (69.5)	162 (69.5)	
Sanitary toilet			0.130
Yes	9 (4.8)	5 (2.1)	
No	178 (95.2)	228 (97.9)	
Kind of toilet			0.763
Latrine with flush	13 (7.0)	18 (7.7)	
Latrine without flush	174 (93.0)	215 (92.3)	
Gender of person who had diarrhea			0.325
Male	118 (63.8)	137 (59.1)	
Female	67 (36.2)	95 (40.9)	
Source of drinking water			0.262
Non improved	60 (32.1)	87 (37.3)	
Improved	127 (67.9)	146 (62.7)	
Source of washing water			0.031
Non improved	178 (95.2)	230 (98.7)	
Improved	9 (4.8)	3 (1.3)	
Source of cooking water			0.315
Non improved	100 (53.5)	136 (58.4)	
Improved	87 (46.5)	97 (41.6)	
Place of wastewater disposal			0.083
Open area around the house	127 (67.9)	176 (75.5)	
Close sewerage system	60 (32.1)	57 (24.5)	
Availability of soap for hands washing			0.413
Yes	80 (42.8)	109 (46.8)	
No	107 (57.2)	124 (53.2)	
Continuous variables			
Age of household head (years)	42.4 ± 14.4 (22–67)	40.9 ± 13.7 (22–67)	0.281
Living in the area (years)	15.2 ± 10.5 (1–44)	15.7 ± 10.5 (1–44)	0.586
Number of rooms in house	2.7 ± 0.8 (1–5)	2.7 ± 0.9 (1–5)	0.524
Number of family Members	6.3 ± 1.7 (3–11)	6.2 ± 1.7 (2–12)	0.577
Age of getting diarrhea	8.7 ± 13.5 (1–62)	21.1 ± 16.7 (1–67)	< 0.001
Number of children less than 5 in family	1.5 ± 0.6 (0–3)	1.4 ± 0.6 (0–3)	0.238
Distance to healthcare center (km)	1.6 ± 0.8 (1–4)	1.7 ± 0.8 (1–4)	0.303

For the continuous variables, the Mean ± SD (min-max) were presented instead of frequency and percentage as in the binary variables

Chi-square test was used to examine the difference between the two groups

To identify the trend of seeking healthcare behavior from professional and non-professional healthcare providers and vice versa. The 421 household’s heads who indicated that there had been an episode of diarrhea within their household were asked about their seeking behavior concerning healthcare professionalism for diarrheal treatment in the last three diarrheal episodes. Approximately 52.6% (221/199) sought health care from a professional health care provider in the first episode of diarrhea, around 33.6% (141/279) sought professional care provider in the second episode, whereas 66.4% sought non-professional healthcare. In last diarrheal episode, the trend of seeking professional care providers quite improved and was in favor of professional providers (44.5%) compared to 55.5% (187/233) who sought non-professional care (Table 5).

**Table 5:** | Healthcare utilization patterns of subjects reporting diarrhea in the Gaza strip

Healthcare provider	First time	Second time	Last time
Professional	221 (52.6%)	141 (33.6%)	187 (44.5%)
Non-professional	199 (47.4%)	279 (66.4%)	233 (55.5%)

### Independent factors associated with seeking non-professional healthcare

Six predictors were generalized for seeking non-professional healthcare for diarrhea treatment among the population of Gaza strip. These factors are a female head of the family (OR = 2.1; 95% CI: 1.1–4), low education level of household head (OR = 4.9; 95% CI: 2.2–8), low monthly income (OR = 4.1; 95% CI: 2.7–7.5), age of getting diarrhea (> 5 years old) (OR = 2.2; 95% CI: 1.1–4.4), living in rural areas (OR = 9.1; 95% CI: 1.9–44.3), and disposal of wastewater in the an open area surrounding the house (OR = 1.8; 95% CI: 1.1–3.1) (Table 6).

**Table 6:** **|** Predictors of seeking care from non-professional healthcare providers among the population of Gaza strip

Variable	Odds ratio	95% CI	*P*-value
A female head of the household^a^	2.1	(1.1–4)	0.018
Low education level of household head^b^	4.9	(2.2–8)	0.001
Low monthly income of household^c^	4.1	(2.7–7.5)	0.001
Age of person who got diarrhea more than 5 years^d^	2.2	(1.1–4.4)	0.001
Living in rural areas^e^	9.1	(1.9–44.3)	0.006
Disposal of wastewater in an open area around the house^f^	1.8	(1.1–3.1)	0.032

The level of measurement for all variables in the table was nominal

The default references used for the variables in the table were:

^a^A male head of the household a

^b^High education level of household head

^c^High monthly income of household

^d^Age of person who got diarrhea less than 5 years

^e^Living in urban areas

^f^Disposal of wastewater in a close sewerage system

## Discussion

In our study, we reported a high prevalence of diarrhea in the Gaza strip (3.8 per 100). Factors related to this prevalence are complex in nature including but not limited to ineffective sewage management and lack access to safe drinking water [18, 19]. The epidemiological bulletin published by the UNRWA indicated a constantly increase in trend of diarrheal incidence between 2010 and 2013 [4]. Diarrhea is more prevalent among young children. The previous finding from the same population was consistent with ours. Kanoa and his colleagues found diarrhea highly prevalent among under 5 years old [20]. Similarly, the global pattern of diarrheal is alike and it is the second leading cause of mortality and morbidity in children less than five [21–23]. However, the burden of diarrhea remains heavy especially in industrialized countries where older ages represent a large portion of their populations [11]. A consensus between the results of our study and the final edition of the population and housing report published by the Palestinian Central Bureau of Statistics was found with respect to the average number of the Gaza’s household’s members which was around 6 members [12].

Risk of diarrhea differs between gender and males showed more risky than females. Similar findings were reported Bangladesh and Sudan [11, 24, 25]. In contrast, Schlagenhauf and his colleagues assessed gender differences in travel-associated disease and found females are more likely to get diarrhea than males [26].

We explored predictors of diarrheal illness among the population of Gaza strip and were living in rural areas, having an unclean kitchen, having a non-sanitary toilet, having latrine without a flush, disposing wastewater in an open area surrounding the home, and washing hands without soap. This finding is consistent with previous studies from Ethiopia, Kenya, and Bangladesh [11, 27, 28]. Knowing the influence of sociodemographic variables on diarrheal illness is vital because it is not only related to the individual case but also societal characteristics, in general, that affect the risk of diarrhea. Previous studies concluded that houses in urban areas and adequate household income affect the ability of families to have access to improved water, sanitation, and hygiene facilities as well as to necessary healthcare that would reduce the risk of diarrhea [29–32].

The education level of mother is seemed to be a significant predictor. Poor educated mother presented a predictor for high risk of getting diarrhea for their children. This finding is persistent with previous relevant studies [33, 34]. The systematic review of Cairncross and her colleagues concluded the importance of hand washing and having a closed sewage system to prevent the incidence and reduce the burden of diarrhea [35].

We explored predictors for seeking healthcare from non-professional provider similar to previous reports. We found low monthly income and living in rural areas were barriers to accessing professional healthcare which is consistent with ex-studies from low-income countries [2, 6, 11, 36]. This is similar to findings obtained from Chowdhury et al. 2015 [11]. We also found that the low level of education of the head of household reduces the chance of family members receiving professional healthcare. Perhaps this is due to the lack of awareness of the importance of receiving professional healthcare in the event of diarrhea illness [11, 37]. Families are unable to afford costs of professional treatments and transportations to access services at primary health centers. Moreover, low educated families are in a hurry to see their children recovered fast, so they seek non-professional healthcare.

This study drew the trend of seeking healthcare behavior from professional and non-professional healthcare providers and vice versa in the last three diarrheal episodes. We found about 47.4% initially sought care from a non-professional healthcare provider. This result could be explained by either because the symptoms of diarrhea were not serious to require professional health care or due to denial of Gaza’s households from accessing professional healthcare because of poverty and rural residency, consequently, people prefer to depend on traditional medicine and intake of medicinal herbs as self-treatment. Therefore, providing low-cost and attainable professional health care for diarrheal treatment could decrease the burden of illness [38–40]. In this study, a transition in seeking diarrhea treatment from professional healthcare to non-professional care and vice versa was observed. This could be clearly interpreted as either because of disparities in the severity of the symptoms of diarrhea each time or due to lack of satisfaction with recovery in the previous diarrheal episode, therefore, they returned to seek another healthcare provider different from its predecessor [41, 42].

This community-based cross-sectional study has many methodological strengths, including the relatively large sample size of 1857 households, the method of interviewed questionnaire for data collection is superior to a self-administered questionnaire, using a cluster random sampling to guarantee representativeness of the sample from the five Gaza’s governorates. Therefore, generalization could possibly be applied to all of Gaza’s areas. Furthermore, the main strength of our study was it is being the first study, which identified the predictors for seeking nonprofessional care for diarrheal treatment. However, it also has some noteworthy limitations. First, many independent factors were not examined for instance: dietary habits, early weaning, seasonal patterns, lack of safe water supply, younger maternal age, and indiscriminate disposal of child feces [43–46]. Second, recall bias and misreporting could happen when we asked about seeking healthcare behavior in the last three diarrheal episodes.

## Conclusions

This study showed a high prevalence of diarrhea particularly among children less than 5 years old. Sociodemographic, economic, water, sanitation, and hygiene factors in the Gaza strip’s households were the predictors of the diarrheal illness and seeking non-professional healthcare for diarrhea illness treatment. Consequently, in the light of study findings, we recommend improving the status of water, sanitation, and hygiene to reduce the incidence of diarrhea and provide a low-cost and attainable professional health care for diarrheal treatment. Future studies to investigate the seasonal trend of diarrhea cases considering water, sanitation and hygiene (WASH) variables that were not included in this study are recommended.

## Data Availability

The datasets generated and/or analyzed during the current study are not publicly available but are available from the corresponding author on reasonable request.

## Abbreviations

CI: Confidence interval
INS: Israeli new shekel
NGOs: Nongovernmental organizations
OR: Odds ratio
PHCCs: Primary healthcare centers
SPSS: Statistical Package for Social Science
UNRWA: United Nations relief and works agency for Palestine refugees in the Near East
WASH: Water, sanitation, and hygiene
WHO: World health organization
